# Impact of Processing and Physicochemical Parameter on *Hibiscus sabdariffa* Calyxes Biomolecules and Antioxidant Activity: From Powder Production to Reconstitution

**DOI:** 10.3390/foods12162984

**Published:** 2023-08-08

**Authors:** Cho Urielle M’be, Joël Scher, Claire Gaiani, N’Guessan Georges Amani, Jennifer Burgain

**Affiliations:** 1 LIBio, Université de Lorraine, 54000 Nancy, Franceclaire.gaiani@univ-lorraine.fr (C.G.); 2 UFR STA, Université Nangui Abrogoua, Abidjan P.O. Box 801, Côte d’Ivoire

**Keywords:** *Hibiscus sabdariffa*, drying process, powder production, structure change, color change, bioactive molecule, stability, antioxidant activity, extractability, reconstitutability

## Abstract

*Hibiscus sabdariffa* is a tropical plant with red calyxes whose anthocyanins, phenols, and antioxidant activity make it attractive to consumers both from a nutritional and medicinal standpoint. Its seasonality, perishability, and anthocyanin instability, led to the setup of stabilization methods comprising drying and powdering. However, its properties can often be altered during these stabilization processes. Treatments such as dehumidified-air-drying, infrared drying, and oven-drying, and their combination showed better quality preservation. Moreover, powder production enables superior biomolecule extractability which can be linked to a higher bioaccessibility. However, the required temperatures for powder production increase the bioactive molecules degradation leading to their antioxidant activity loss. To overcome this issue, ambient or cryogenic grinding could be an excellent method to improve the biomolecule bioavailability and accessibility if the processing steps are well mastered. To be sure to benefit from the final nutritional quality of the powder, such as the antioxidant activity of biomolecules, powders have to offer excellent reconstitutability which is linked to powder physicochemical properties and the reconstitution media. Typically, the finest powder granulometry and using an agitated low-temperature reconstitution media allow for improving anthocyanin extractability and stability. In this review, the relevant physicochemical and processing parameters influencing plant powder features from processing transformation to reconstitution will be presented with a focus on bioactive molecules and antioxidant activity preservation.

## 1. Introduction

Consumers are progressively giving more credence to minimally processed and natural products that naturally contain biomolecules with interesting properties such as plant-based products. This market trend could be explained by their attractive characteristics including the nutritional quality and the health properties. Such characteristics are attributed to the plant-based product richness in vitamins, minerals, and some compounds essential to the functioning of the human body [1] similar to the antioxidant biomolecules. People’s preference for natural products is also a way to guarantee their quality since the health effects of synthetic food ingredients have long been controversial. As a result, attention is also being paid to the substitution of synthetic antioxidant products with natural antioxidants [2] or antioxidant-rich natural products, even though the current market offers mostly synthetic vitamins and antioxidants.

To raise awareness of healthy foods, FAO (Food and Agriculture Organization of the United Nations) has declared 2021 as the year of fruits and vegetables, including plants that are consumed fresh or minimally processed (washing, peeling, slicing, packaging, freezing, drying, etc.). This sensitization relates to major societal issues such as biodiversity, diversified and healthy diet, food safety, and reduction of food waste of perishable products. Paradoxically, fruits and vegetables are included in the top two groups of food losses, with 22% of losses worldwide, after roots and tubers (25%) [3]. This is explained by their extremely perishable nature, due to their water content, which makes them an ideal reaction medium for degradation reactions such as microbial growth. To lower the food loss rate, a drying process is often suggested, which reduces the water content to a critical value leading to limiting the microorganism development. Moreover, conversion to powder is a complementary space-saving option when considering the transportation of these products. Indeed, fruit and vegetable production is often limited to the production regions, exportation is therefore essential so that the majority of the population can enjoy their health and nutritional benefits.

*Hibiscus sabdariffa* (hibiscus) calyxes fit well in this context because this perishable plant naturally contains minerals, biomolecules such as polyphenols, flavonoids, and specifically anthocyanins [4,5,6]. On the one hand, this composition makes the hibiscus calyxes particularly attractive thanks to their red color and their sour-fruity taste [7], which are the first criteria for the choice and acceptability [8] of the final product by consumers. Attention must therefore be paid to these sensory properties given some foods with interesting biomolecule contents sometimes have an unattractive appearance (color, smell, shape) for consumers [9].

On the other hand, polyphenols and anthocyanins promote an important antioxidant activity, which attributes health benefits to *Hibiscus sabdariffa* calyxes, and makes them highly coveted products [7]. Hibiscus therefore has real economic and health potential, which can only be exploited if it is well preserved from production to processing. Adding value to hibiscus also involves developing processes that will facilitate the extraction of key biomolecules, make the most of their antioxidant properties, and facilitate the end products uses for consumers.

In this context, drying and processing the calyxes into powder allows for stabilizing the hibiscus calyx and facilitating access to its health benefits by improving the bioavailability and biomolecule accessibility, provided the process is well mastered [10,11]. Indeed, a reduced particle yields a higher specific surface area improving the biomolecule extraction. To be appreciated, and for good economic return, hibiscus powder has to meet the best organoleptic quality, physicochemical (fines powder, low water activity), flowability, and reconstitutability properties (necessary to use powders), and mainly antioxidant activity. However, the biomolecule sensitivity remains challenging due to their degradation during the stabilization processing [12,13,14,15]. The biomolecule stability is therefore an important criterion for the preservation of the initial properties (antioxidant activity) of products to take advantage of the health benefits and nutritional quality of hibiscus. 

This review will first report the growing conditions and hibiscus composition that justify the use of stabilization processing (drying methods). Secondly, the powder production (drying and grinding or spray-drying) and fractionation processes will be discussed, with particular reference to their potential impact on the product’s original properties (structure, color, chemical composition, and antioxidant activity). Finally, the way in which powders could be used, in terms of their suitability for reconstitution will be addressed.

## 2. *Hibiscus sabdariffa* Plant

### 2.1. Production, Growing, and Culture

**Figure 1:**
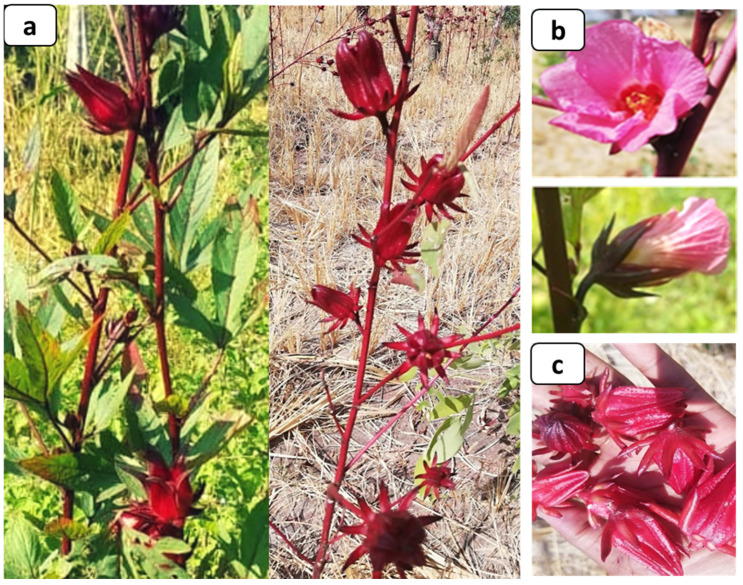
*Hibiscus sabdariffa*, (**a**)—plant, (**b**)—flowers, (**c**)—calyx.

*Hibiscus sabdariffa* is a herbaceous plant of the Malvaceae family [16] cultivated in many tropical and subtropical countries [4,5,16,17,18]. The different parts of the plant are illustrated in Figure 1.

From this Malvaceae family, two botanical types of *Hibiscus sabdariffa* are distinguished: *H. sabdariffa* variety sabdariffa and *H. sabdariffa* variety altissima. The latter rich in fiber is used as a substitute for jute, coarse sacking [16]. Two types of *Hibiscus sabdariffa* calyxes exist, red and the other white, with similar compound content except for anthocyanins. Many varieties of *Hibiscus sabdariffa* with red calyxes exist. For example, Vimto (the most appreciated), Koor, CLT 92, Thaï, Burkinabe, Yoump, and Violette are all Senegalese varieties with specific features [4]. The world’s best crops come from Sudan, the most important African producer [8]. Different vernacular names are given to the red calyxes such as Jamaica flowers in Central America, Krachiap Daeng in Thailand, sorrel in Guinea, bissap in Senegal and Ivory Coast, karkade in north Africa, ngai-ngai in Central Africa, Folere in Cameroun [4,5,17,18,19]. 

**Figure 2:**
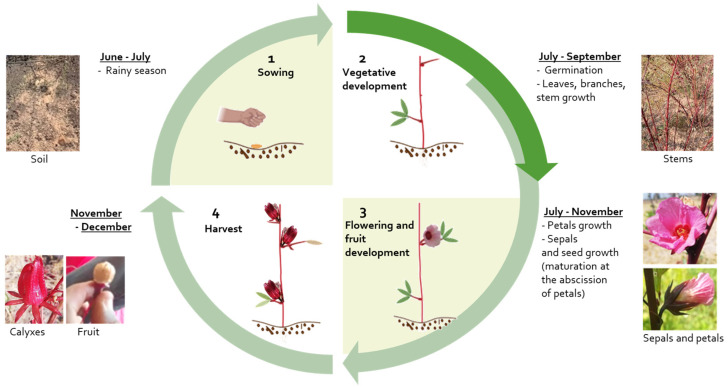
The growth stages of *Hibiscus sabdariffa* plant.

Hibiscus plants growth requirements are: a minimum temperature of 20 °C, drained soils, although they can grow in poor soil, and reach maturity after 4 to 8 months [4,7,16,20]. The growing phase may be divided into four steps (Figure 2). The first step is sowing which is conducted during the rainy season from June to July in Ivory Coast or July to August in Senegal [4]. Secondly, the vegetative development including the stem, branches, and leaves growth, begins. Simultaneously, petals grow, unfurl and fruits develop, highlighting the beginning of the third step of calyx development. During this period, petals close until their abscission, which marks the ripening of fruits and calyxes, and the beginning of the last step which is harvesting. The tender and fleshy red calyxes are collected 2 or 3 weeks after flowering by cutting them at their base [4]. Calyxes harvesting occurs between November and January in Senegal and Ivory Coast but it depends on the rainy season. Calyxes are then shelled, generally sun-dried, packaged, and stored.

### 2.2. Chemical Composition

**Figure 3:**
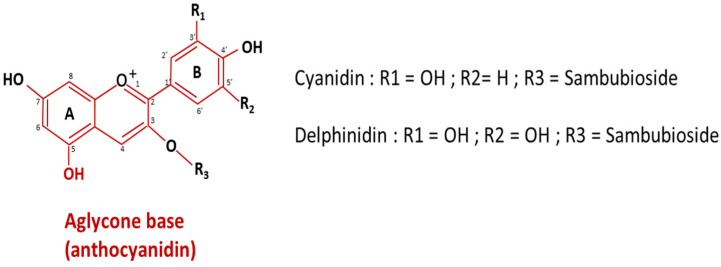
Chemical structure of major anthocyanins in *Hibiscus sabdariffa* calyx [5].

The composition and physicochemical properties of *Hibiscus sabdariffa* calyxes vary according to varieties, origin, and culture method. Fresh calyxes are sources of water, proteins, fibers, and carbohydrates [4]. Studies reported that fresh calyxes roughly contain 80 to 90 g/100 g water content [4,21], 0.9–17.9 g/100 g protein, 0.1–3.9 g/100 g lipid, 2.3–12 g/100 g fiber, 3.3–12.3 g/100 g carbohydrates with 40% of glucose [4,16]. In addition, hibiscus calyxes are a source of mineral elements (K, Ca, Mg, Fe, Mn, Zn) and organic acids (malic, citric, stearic, tartaric, ascorbic, succinic, oxalic acids) [4]. Succinic and oxalic acids represent together 76% of all organic acids [4,16,22,23,24,25]. The average ascorbic acid content is about 72 mg/100 g [4] which is higher than orange juice ranging from 49 to 54 mg/100 g [26,27] and 2.5 and 3 times higher compared to that of blackcurrant and grapes respectively [12,28]. This wide range of acids imparts *Hibiscus sabdariffa* calyxes an acidic pH < 3 [4,17,29] and favors the dissolution of hibiscus minerals [30,31]. The red color of hibiscus calyxes is due to the presence of the anthocyanin molecules that are made up of two parts: a carbohydrate part linked to an aglycone base called anthocyanidin which has two aromatic cycles A and B, and an oxygen-containing heterocycle (Figure 3). The anthocyanin content (150–1500 mg/100 g) [4,16] can be about three times higher than that of black grapes (50–300 mg/100 g) [32]. Two major anthocyanins (Figure 3) have been identified, delphinidin-3-sambubioside or delphinidin-3-xylosylglucoside and cyanidin-3-sambubioside or cyanidin-3-xylosylglucoside, respectively 71 and 29% of all anthocyanins and two minor, delphinidin-3-glucoside and cyanidin-3-glucoside [4,5,12,25,33]. In addition to its red color, the strong antioxidant activity of anthocyanin increases the interest in exploiting these molecules. Indeed, anthocyanins are the main molecules responsible for the antioxidant activity of the hibiscus calyxes [34]. Moreover, *Hibiscus sabdariffa* calyxes contain phenolic compounds including protocatechuic acid and catechin a type of natural phenol presenting antioxidant properties [12,16]. 

According to the calyx composition, particularly the high anthocyanin content, antioxidant activity, and sour-fruity taste makes the *Hibiscus sabdariffa* calyx a very coveted plant part in several fields of activity such as cosmetics, pharmacy, medicine, and in the food industry.

### 2.3. Food and Medicinal Uses

**Figure 4:**
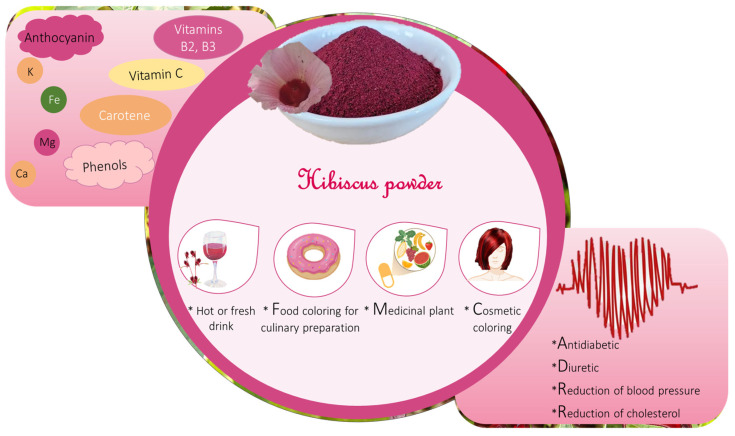
Composition of *Hibiscus sabdariffa* calyxes, their food and medicinal uses.

In medicine as well as in the cosmetic or food industries, hibiscus calyxes are processed to obtain hibiscus products or to extract molecules of interest such as anthocyanin (Figure 4).

*Hibiscus sabdariffa* calyxes are used in the food industry mainly to produce hot or fresh drinks, fermented beverages, and wines. Cocktails, syrups, and fruit salad are also made with hibiscus calyx juice. Hibiscus calyxes or powders are added as natural coloring agents to have red coloration in cooked foods, pastry foods, puddings and cakes, chocolate [8,16,18,34]. The United States of America and the European Union classify anthocyanin as a food colorant under the category of fruit (21 CFR 73.250) or vegetable (21 CFR 73.260) and as a natural coloring agent under classification number E163 [35]. In addition, delicious jelly and jam, ice cream of hibiscus calyx are manufactured and appreciated all over the world [4]. 

*Hibiscus sabdariffa* calyx is also known as a medicinal herb due to its composition and special features. Indeed, *Hibiscus sabdariffa* calyxes are used for their diuretic, febrifugal, anthelminthic, antimicrobial, antidiabetic, hypotensive, anti-inflammatory, hepatoprotective, and hypocholesterolemic activities and to stimulate the intestinal peristalsis [16,19,33,36,37,38,39,40,41,42,43]. Moreover, anthocyanin molecules can reduce the risks of coronary heart disease due to their antioxidant properties [4,5,33,37].

### 2.4. Interest in Stabilizing Hibiscus sabdariffa Calyx

The previous parts underlined the fact that the main interest in exploiting hibiscus calyxes lies in their composition and particularly in their richness in anthocyanin, which is responsible for their red color and antioxidant activity. The product appearance including its color is the first criterion of choice for the customer or consumer, followed by the nutritional quality, more specifically the richness in antioxidants and their health-promoting power [8]. Conscious of the impact of their food on their health, consumers are becoming more demanding, and rightly so. Consumers are also increasingly concerned about the impact of their consumption on the environment (the non-overexploitation of land, the biodiversity) but also about the fair remuneration of farmers and producers. To meet the requirements of this market, but also to avoid food waste, it is necessary to set up stabilization processes that will make it possible to:

- Alleviate the problem of the seasonality of hibiscus;
- Make the product available throughout the year;
- Ensure a long shelf life;
- Facilitate transport from a producing region to an importing region;
- Make the product accessible;
- Facilitate handling and use of the product;
- Facilitate the extraction of compounds of interest such as anthocyanin.

## 3. Stabilizing Processes and Impact on Products

The nutritional value, organoleptic qualities, and medicinal benefits attributed to *Hibiscus sabdariffa* calyxes and other plants or vegetables make them interesting and useful products. Numerous processing methods have been investigated and set up to develop stabilized products including drying, extraction, evaporation, and powdering.

### 3.1. Drying

Drying is a way to remove partially or completely water from a material. In this way, many perishable plants, fruits, and vegetables such as *Hibiscus sabdariffa* calyxes are dried, to reduce water content for better preservation, also to overcome the seasonality issues. The reduction of water content is a good way to extend the shelf life, to achieve lightweight and low volume for easy handling and transport. The desire for safer products leads to studying and appreciating different drying technologies from old to advanced methods depending on the product properties, and the available resources (financial, devices, renewable energy, etc.).

**Figure 5:**
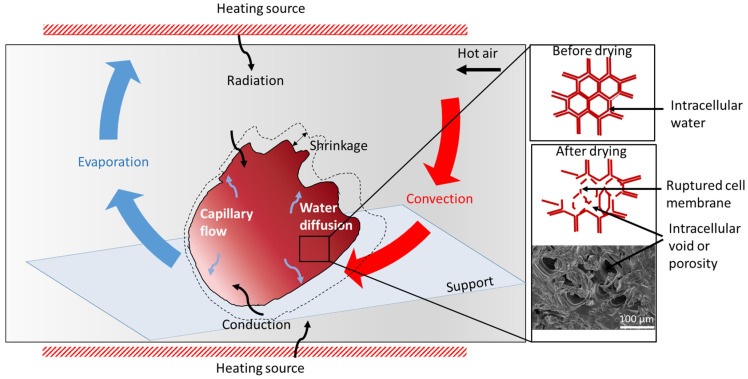
A representation of a thermal drying process with a focus on hibiscus calyx and is cellular structure.

The elimination of water during drying is the result of the simultaneous heat and mass (water) transfer between the product and its drying environment [44]. Heat is transferred from the drying environment to the product in different ways (Figure 5) depending on the drying type, comprising convection, radiation, conduction, microwave, radio-frequency, and Joule (ohmic) heating [44,45]. The supplied heat allows increasing the material temperature, inducing a water phase change from water to vapor (due to latent heat at constant temperature) and activating molecular movement. Water is transferred in the opposite direction from the interior to the material surface by capillary flow (liquid) and/or diffusion (liquid or vapor) and diffuses from the surface to the drying medium (vapor) [44]. The most common drying treatments of *Hibiscus sabdariffa* in addition to solar drying are hot air drying and dehumidified air-drying all resumed in Table 1.

#### 3.1.1. Sun-Drying

The solar drying process consists of heating the product by radiation, and convection using the sunrays and the air as a heat-carrying fluid to induce the evaporation of material water. To a lesser extent, heat is transferred by conduction through the support drying. Therefore this drying process depends on extrinsic factors such as the weather conditions (sun position, duration of sunshine, temperature, humidity, rain, and air velocity) (Table 2) and intrinsic factors of the product such as the specific surface area, chemical composition, and physical structure (porosity, density, size and shape) [12,47]. Solar energy is a fundamental energy for tropical and sub-tropical regions since it is accessible, renewable, and free of charge. 

#### 3.1.2. Hot Air Drying

This process is commonly used in industries because of the facility to handle, the low investment, and the cost (Table 2) compared to improved technologies [48]. Hot air drying is carried out in an oven with convection heating, where warm air is used to remove water from products. Contrary to sun-drying, the water content of the dried products is less variable due to a controlled and confined environment that is independent of weather [12]. High drying temperatures lead to higher drying rates and water content loss. 

#### 3.1.3. Dehumidified-Air-Drying

This process is an advanced technology that consists of drying by keeping the dryer at low temperature and humidity. The principle is to extract the excess humid air exchanged with the moist product, which is placed inside the drying chamber. Dehumidified air-drying is not dependent on weather factors and can maintain the optimum drying conditions [12,49]. This continuous drying process solves the problem of rehydration overnight upon solar drying. Tham et al. [12] showed that dehumidified-air-drying allows obtaining the highest drying rate of *Hibiscus sabdariffa* flowers compared to oven-drying, solar with intermittent heat pump drying, and solar drying. Indeed to remove moisture on the solid surface, a greater drying force is created by reducing the air’s relative humidity. The drying time is then shortened in comparison with solar drying and solar with intermittent heat pump drying respectively. 

#### 3.1.4. Microwave Drying

Microwave drying applies fast changing electromagnetic field that implies the repeated rotation of water molecules resulting in heat production from the inner part of food. Therefore, this method induces the sudden water migration and evaporation [50,51]. Compared to several conventional drying methods (e.g., solar drying, hot air drying), this method is based on a short drying time, high drying rate and quality retention [52]. Upon microwave drying, the product could require to be constantly in rotation because of the non-uniform heating.

#### 3.1.5. Infrared Drying

This technique relies on electromagnetic radiation, which depends on the temperature source [51,53]. The heating energy is directly transferred from the source to the product. This could be the principal reason for its efficiency since the heat loss could be reduced, and the product heating is uniform contrary to microwave drying [51,54]. The drying rate is higher than the conventional methods such as hot-air-drying, and solar-drying. This drying rate is even improved when the treatments are associated. For example, infrared and microwave drying or hot drying reduced the drying time by up to 76% compared to pure hot air drying [55]. Drying time reduction is also reported by combining infrared radiation with a heat pump, freeze drying, and intermittent microwave drying [51].

#### 3.1.6. Impact of Drying Processes

**Figure 6:**
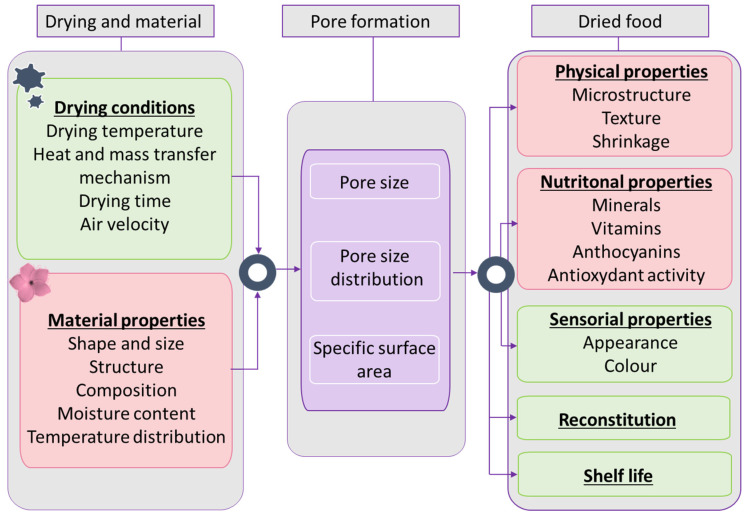
Interactions between drying parameters, material properties, pore formation, and dried food properties.

Physical modifications may occur during drying, such as pore formation, shrinkage, and color change. Porosity is due to the intercellular voids (spaces) which appear upon the drying treatment. The water removal from the intercellular region leads to the loosening of intercellular bonds and the progressive separation of cells from each other and the removed water is substituted by air [56,57]. The material porosity is linked to drying conditions and the material characteristics (Figure 6) [56]. Highly porous structures are observed when increasing the drying temperature, sample size, and decreasing the drying time [58]. Fast drying causes a more rapid water removal from the material surface than the interior so that internal stresses are induced resulting in tougher and porous material [56,59].

The porosity could be an appreciated parameter, as this microstructural characteristic is required to improve sensory quality [56] and rehydration properties of final products [10].

Shrinkage is determinant in the definition of bulk density. The more the products shrink, the lower the volume, and the higher the bulk density. Similar to other plants, the epidermis of hibiscus calyxes are formed of cells made up of lamella, wall (primary and secondary), and plasma membrane. The cells of the epidermis of hibiscus calyxes are particularly well-organized, tightly packed, and thick-walled [13]. The cell wall, plasma, and the vacuolar membrane are broken down under the effect of drying temperature. Progressively the calyxes fold, and become deformed, leading to shrinkage [13]. Indeed, the cell liquid exerts a pressure called turgor pressure on the cell membrane keeping the cell in a state of elastic stress, which ensures the stability of the material shape, texture, firmness, and crispness. During drying, the turgor pressure is lost and causes the collapse of herb or plant products [56,60,61]. The final structure of the dried calyxes therefore depends on the type of drying applied. For example, freeze-drying is a process that requires a freezing step during which the water included in the calyxes is converted into ice and then sublimated. During such a process, it was observed that the structure was preserved, and the surface was smooth such as fresh calyxes. In contrast, the structure of sun-dried calyxes was the most deformed and shrunk. The temperature and the drying duration may be the reasons [13], but also the material thickness, and the drying rate. Higher drying rate, temperature, and air velocity induce more structure deformation [60].

The color change is one of the prevailing physical effects resulting from drying. On the one hand, the physical deformation (collapse and porosity) submitted by the material during drying could change the optical properties such as the scattering, reflection, transmission, and absorption of visible light. This, therefore, modifies the color parameters [56].

On the other hand, dried products can undergo color change due to the pigment concentration induced by removing water. The color modification is also depending on the drying treatment applied (Table 3). Upon drying, the colorimetric parameters (lightness (L*), redness (a*), yellowness (b*), and total color change (ΔE) comprising L*, a*, b*) of calyxes could change. Temperature is the wider parameter influencing the coloration. For instance, solar drying that requires higher temperature and drying time induced higher total color change (7.28 ± 2.67) than oven-drying (6.57 ± 2.02) and solar with intermittent heat pump drying (6.47 ± 2.78) [12]. Based on lightness, microwaved apricots showed better preservation (20.1–24.4% reduction) unlike the oven-dried apricots (30–34% reduction) at 60 and 70 °C [62] probably due to their longer drying time (Table 2). Dissimilarly, Baysal et al. (2003) [63] reported good coloration results for carrot and garlic microwave or hot air drying instead of infrared drying. The radiation and temperature required for infrared drying probably lead to color loss. The color change could also be justified by the non-enzymatic browning reaction, leading to more reddish and yellowish hibiscus calyxes [12]. In addition, a high level of oxygen can stimulate the browning reaction which induces an increase in saturation (the chroma value) [12,64], and a decrease in lightness [62].

Moreover, the oxidized components could react with antioxidant molecules such as anthocyanin, giving rise to colorless or brown products [64]. This could consequently amplify the material discoloration due to pigment losses. The color modification is also due to the degradation of chemical compounds upon drying.

**Figure 7:**
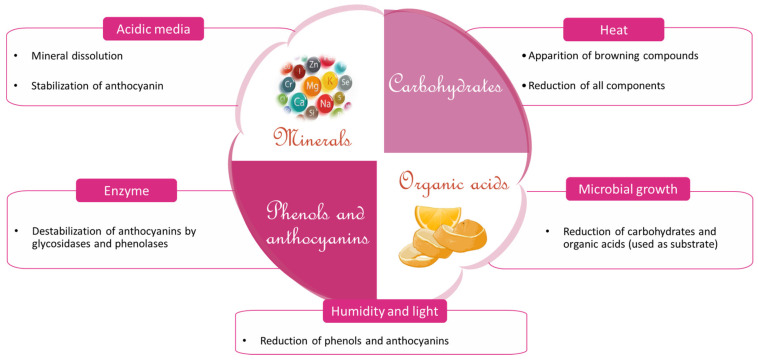
Sensitivity of constituents of *Hibiscus sabdariffa* calyxes.

This biomolecule loss therefore depends on the drying method, the drying conditions (temperature, air velocity, drying time), and the intrinsic material properties [48,65,66,67]. Indeed, molecules that contribute to material pigmentation and are responsible for the antioxidant activity for example phenolics (protocatechuic, catechin acids), and anthocyanin compounds in hibiscus calyxes, chlorophyll, and carotene are differently heat sensitive (Figure 7 and Table 3).

These molecules became less stable during heat treatment, leading to their decomposition therefore to their losses [12,29,68]. For example, reductions by 15.3% and 36.9% of hibiscus phenol and anthocyanin contents during solar drying treatment [29] (Table 3) were observed.

Bioactive components (e.g., phenol molecules and anthocyanins) are also subjected to degradations when increasing the drying temperature [12,54,66]. When hibiscus calyxes were oven-dried at 60, 80, 100, and 120 °C until the water content was below 8%, Nguyen and Chuyen [46] observed an important decrease in phenol content. Nevertheless, the phenol content at 80 °C was the highest highlighting the beneficial effect of the short time required at this temperature. Comparing the drying temperature of 80 °C to 60 °C, the latter was coupled with a long drying time leading to longer exposure to high temperature, light, and oxygen, which resulted in greater degradation of phenol molecules. This choice of temperature depends on the interested biomolecule to study. According to the results of Sánchez-Feria et al. [29], a good preservation of anthocyanin molecules was observed at 50 °C and 70 °C. At 60 °C, an anthocyanin loss was observed. When considering simultaneously the preservation of phenol molecules, anthocyanins, and organic acids, they suggested 70 °C as the best temperature.

**Figure 8:**
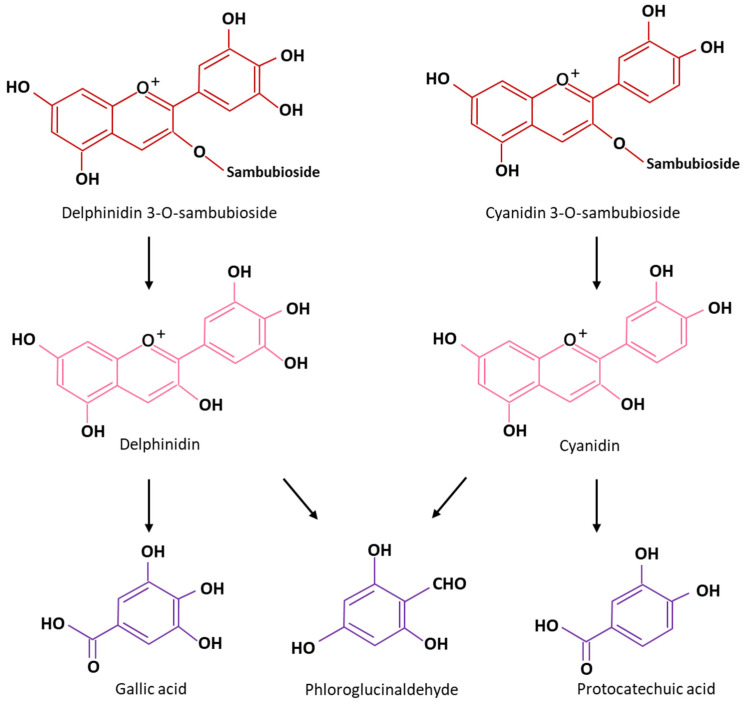
Mechanism of thermal degradation by scission adapted to *Hibiscus sabadariffa* anthocyanins [5].

Accordingly, hibiscus anthocyanins subjected to heat and increasing temperature undergo thermal degradation. One of the wider consequences is the formation of a phenolic acid arising from the B-ring and an aldehyde resulting from the A-ring (Figure 8) [5,69,70].

These observations highlight the thermal sensitivity of biomolecules and show the need for soft drying [12,48,67,71] such as dehumidified-air-drying, and to make compromises between drying time and temperature. The efficiency of water transfer also depends on air vapor saturation, and its lowering will favor faster drying.

The biomolecule loss tendency during drying was also supported by Albanese et al. (2013) [62]. Against all expectations, microwave-drying, despite its lower drying time presented a lower β-carotene retention (60%) compared to oven-drying (80%). Inversely, for better preservation of phenols and flavonoids in hibiscus leaves and their antioxidant activity, microwave drying was often preferred to vacuum drying and oven drying [72]. These results are in concordance with the studies of Jin et al. (2018) [55], who showed better phenol and flavonoid contents during infrared drying, followed by hot air drying and microwave drying. This tendency evidenced the interest in considering the material, optimal power condition, targeted biomolecule sensitivity for each process, and the oxidation risks. Studies reported better ways to preserve biomolecules and therefore the nutritional quality by the drying process combination method. Working on sour cherries, Jin et al. (2018) [71] showed better nutritional quality by associating vacuum drying with microwave-drying at 480 W, then at 120 W. Other combinations such as microwave-drying and oven-drying, freeze-drying, infrared-drying, shade drying permitted to reduce the microwave treatment damage [51,73].

In addition, molecule losses could occur during fermentation of the organic acids by the natural microflora (Figure 7). This fermentation reaction is favored by the long-time of drying (and relatively low temperature) and can be observed during solar drying of plant material such as hibiscus calyxes. Inversely in hot air drying, an inactivation of the microflora was observed for temperatures ≥ 50 °C for hibiscus for example. This temperature coupled with short exposure time (6 to 8 h for hibiscus calyxes), favors better retention of organic acids [29].

As mentioned above, the optimal drying conditions and parameters are found in the function of the work target. From this fact, one or several biomolecules are selected to be preserved during the process. Nevertheless, in the example of biomolecule-rich material, more than one bioactive molecule is often necessary to be protected, to ensure the best antioxidant activity of the final product.

### 3.2. Powder Production

Powder production improves the functionalities of plants, herbs, spices, vegetables, and fruits such as hibiscus calyxes by increasing the bioavailability of components, as well as antioxidant activity [3,59,74,75]. This process facilitates the handling and storage by reducing the product volume.

#### 3.2.1. Liquid Conversion into Solid Material

##### Spray-Drying

Spray-drying is the transformation of a concentrated liquid into a dried particulate form by spraying the feed into a hot drying gas. Spray drying involves entrainment. When a wet product is placed in a sufficiently hot and dry gas, a temperature and partial water pressure gradient spontaneously occurs leading to a heat transfer (from the air to the product), a reverse water transfer occurs due to the difference in partial water pressure between the air and the surface of the product. The gas serves as both a heat transfer fluid and a carrier gas for the elimination of water vapor [74].

Spray drying is the most applied process to obtain powders and encapsulates pigments as anthocyanin [21,75,76]. Indeed, it is a good preservation method of biomolecules as well as electrostatic spray drying and nano spray drying [77,78,79]. In addition, *Hibiscus sabdariffa* extracts are spray-dried to have instant powders. To achieve this processing, it is worth noting that spray-drying necessarily comes after an extraction step and ideally, after a concentration step as well, because it reduces the energy consumption, therefore, the energy cost of spray-drying [80].

Physical impacts. Spray-drying requires high inlet temperatures to remove water from materials in a short time (Table 2). These temperatures may be higher than the glass transition temperature impairing the powders, particularly those composed of amorphous particles and sugar-rich materials.

Temperature rise may lead to stickiness, responsible for powder caking, and powder adhesion to the dryer surface (Table 3). Indeed, the increase in temperature could induce the passage of amorphous particles into sticky particles (from a glassy to a rubbery state) when the glass transition temperature (Tg) is reached and/or exceeded [81,82]. For spray dried hibiscus, Langrish and Chiou [83] found a Tg ranging from 50 °C (water content = 10.74 g/100 g) to 73 °C (water content = 8.30 g/100 g). In addition, some materials may reach their melting point causing adhesion to the dryer surface [83]. This phenomenon is common to products rich in carbohydrates or lipids, leads to a loss of powder, and results in lower yield [81,82].

Chemical impacts. Components such as anthocyanin molecules, and ascorbic acid are not as stable and could be altered. Eroğlu et al. [81] assessed a loss of 36.9% and 49.2% anthocyanin and ascorbic acid compared to raw materials, in instant hibiscus blended rosehip powders. In addition, some reactions may occur and result in the formation of new products during spray drying [81]. Analyzing *Hibiscus sabdariffa* calyx powders, Gonzalez-Palomares et al. [21] and Ramírez-Rodrigues et al. [19] detected furfural molecule which is a product of the non-enzymatic reaction, furanic linalool oxide, cis-linalool oxide, eugenol molecules. The formation of these molecules and the loss of initial molecules means that some degradations of initial products and Maillard reaction occur during the process due to the temperature and the presence of oxygen [21,84]. Increasing the temperature is correlated to the increase of these compounds and the loss or degradation of the initial molecules including anthocyanin, and acid ascorbic. The direct consequence is a reduction in the antioxidant activity [81,85]. Carrier agents such as maltodextrin, Arabic gum, pectin, carboxymethyl cellulose, and whey protein may be added to the extracts before spray-drying to encapsulate biomolecules and avoid their loss [81,84,86,87]. However, the concentration of the carrier agent could affect the bioavailability of compounds of interest. For example, Eroğlu et al. [81] highlighted for hibiscus blended rosehip powder, a reduction in ascorbic acid contents with the increase in maltodextrin although the product yield increased.

##### Freeze-Drying

Freeze-drying is another drying method where the material is frozen and then water is removed by sublimation. It differs from the spray drying process that removes the water content from the product by entrainment. Small parts of the material are rapidly frozen to produce ice crystals, then the surrounding pressure is lowered (below 610 Pa), and the ice sublimates when heat is slowly applied to the frozen material [88]. In liquid foods that do not have a cellular structure, slow freezing is used to form a network of large ice crystals. Channels formed by the sublimed ice allow the vapor to escape more quickly than in solid foods [88]. Freeze-drying requires low temperatures, it is successful for most foods and recommended for heat-sensitive products [88,89]. However, it is an onerous drying method up to five times more expensive than conventional drying (Table 2), due to the necessity of previously freezing the sample, followed by ice sublimation at low pressures [84,88]. This method is typically employed for high-added-value products.

Physical impacts. The freeze-dried hibiscus extracts studied by Duangmal et al. [35] became flakes, with a better-preserved and appealing red color for consumers (Table 3). They observed a total color change (ΔE) of only 1.9 ± 0.1 and explained this modification by the degradation of anthocyanin (about 5%) which was correlated to lightness and chroma. Chroma value has been proven to be a good indicator of anthocyanin content due to the strong correlation to this molecule content [35,90]. The color change of freeze-dried hibiscus powder is delayed when using carrier agents such as maltodextrin [35].

Chemical impacts. The great capacity of freeze-drying to preserve the product quality such as biomolecules, smell, and the flavor was reported by several authors including Fellows [88], Duangmal et al. [35], and Gong et al. [67]. Freeze-drying was applied to produce cabbage powder, showing a higher vitamin C, about 1.7 times greater than hot air drying processing [67]. In addition, Duangmal et al. [35] studying freeze-drying of hibiscus extract (water—95% ethanol as solvent) under 0.05 hPa vacuum for 15 h, assessed a high anthocyanin retention with 95.08 ± 5.18%. Water activity plays a non-neglected role in the stability of anthocyanin [35,91,92]. Duangmal et al. [35] obtained the highest hibiscus anthocyanin stability at the lowest water activity (a_w_ = 0.115) for the freeze-dried extract with maltodextrin (3 g/100 mL). This is explained by the reduction of the reactant mobility due to the addition of maltodextrin which might impact the product hygroscopicity [35]. In agreement with this study, Thakur and Arya [92] reported a loss of blue grape anthocyanins with high water activity. Freeze drying remains preferable to microwave drying followed by vacuum drying because of its best biomolecule preservation and antioxidant activity [72].

Furthermore, after freeze-drying, special attention must be paid to the biomolecule degradation during storage. To overcome this issue several studies investigated the addition of stabilizers in extracts and evaluated their effect on the freeze-dried product during storage. In the case of *Hibiscus sabdariffa* for example, the effect of some stabilizers including maltodextrin, trehalose, pullulan, and whey protein isolate on the anthocyanin stability was studied [35,84,93]. The addition of maltodextrin and trehalose favored the stability of hibiscus anthocyanin during storage at 30 °C for 105 days expressed in terms of half-life (the time needed for 50% degradation of anthocyanins) [35]. Duangmal et al. [35] recorded a half-life (t_1/2_) of 92, 97, 247 days for freeze-dried hibiscus extract, freeze-dried hibiscus extract with trehalose (3 g/100 mL), and maltodextrin (3 g/100 mL). The increase (from 2 to 3 g/mL) in trehalose concentration induced a higher loss in anthocyanin content in contrast to maltodextrin which provided better stability of anthocyanin during storage [35]. Indeed, in any medium (liquid or not), maltodextrins bind to molecules in contrast to copigmentation which occurs in aqueous conditions [94]. Dextrins react with the flavylium cation derivatives in the extract giving complexation products that prevent the anthocyanin transformation in other less stable forms [95].

#### 3.2.2. Size Reduction by Dry Grinding

The dry grinding process consists of splitting a product into small pieces after applying mechanical stress using a special device. During grinding, material overcomes deformations leading to breakage. The first deformation may be elastic, in which case the material can be restored to its original shape [96]. With the rise of the applied stress, the material may reach its yield strength. Exceeding this limit, the deformation is termed plastic deformation meaning that the material cannot regain its initial shape. The plastic deformation continues with additional applied stress and reaches the breaking point, therefore the material breaks [96]. Several authors, Chamayou and Fages [96] and Hulin [97] reported that a broken material using low energy and without the elastic deformation step is called brittle material. Chamayou and Fages [96] named malleable materials, products broken after high deformations, mainly plastic deformations. The intermediate class between brittle and malleable is semi-brittle materials.

**Figure 9:**
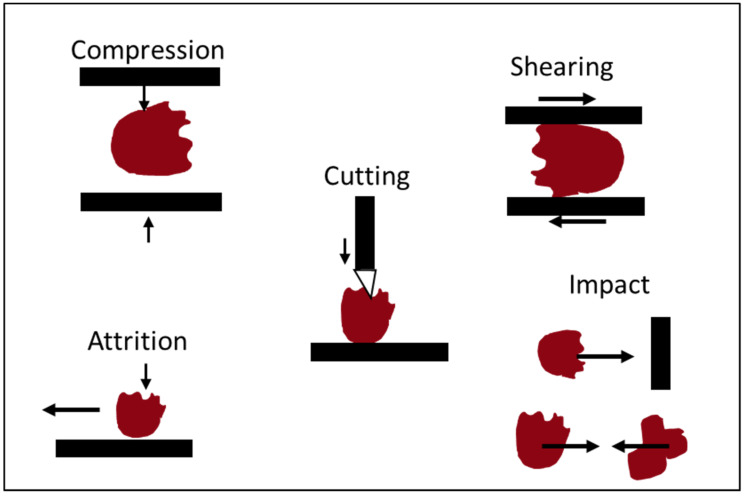
Solid fragmentation modes applied to hibiscus calyxes. The powder particle size depends on the intrinsic parameters of products as physicochemical composition and the structure of materials. It depends also on the extrinsic parameters including the device, the system used to break, and the conditions of grinding such as temperature, relative humidity, and grinding speed.

Moreover, the grinding mode influences the fracture of materials and varies with the grinder. The different fragmentation modes are compression, cutting, shearing, attrition, and impact crushing (Figure 9).

Materials with high water content, fibers, and/or sugar content such as *Hibiscus sabdariffa* calyxes are difficult to grind unlike products rich in minerals, fat, and proteins, and therefore need high energy to be ground [98,99,100].

Dry grinding should be preceded by drying for products with high water content to enhance the grinding process and obtain finer powder [101]. During the grinding process, the energy used to grind may be converted into thermal energy, increasing the temperature of the mill, which in turn heats the ground product. This temperature rise may cause degradation of the molecules retained in the matrix and modify the functional properties according to the material’s thermal sensitivity. In that sense, other grinding methods have been developed to maintain the temperature or to avoid heating during the fragmentation. Some cold grinding methods implemented, allow to maintain the process at the optimum temperatures, including cryogenic grinding, grinding with water cooling, and grinding with liquid nitrogen cooling [48,102,103,104]. In addition, the risk of browning may be reduced by applying vacuum grinding [105].

It can be seen that authors mainly applied the ambient grinding of *Hibiscus sabdariffa* calyxes as a prerequisite, powders are then used to facilitate further processes such as anthocyanin or phenol extraction, rehydration, or encapsulation [11,106,107,108]. Indeed, a reduction in the size of calyxes from 2 cm to 150-85 μm resulted in a considerable reduction in extraction time from several hours to less than 10 min or even 30 s for finer particles with an increase in yields [4,10].

Physical impacts. The increment of temperature during the grinding may impair the powder color by leading to changes in redness, yellowness, and lightness (coloration parameters) [102,104]. Grinding-induced powders obtained at room temperature can be heterogeneous, with different populations unlike the homogeneous powders obtained by grinding at low temperature [11,48,102,104]. A homogeneous population of large particles is more conducive to good flowability than a heterogeneous population because the fine particles fit into the interparticle porosities preventing their flowability [109]. Singh et al. [104] studying ball milling of king chilli at ambient temperatures of 30 ± 2 °C and low temperature—90 ± 3 °C, observed 27.56% finer powder at low temperatures compared to ambient grinding. Indeed, at cryogenic temperature particles were more breakable (below the glass transition temperature) allowing to have smaller milling-induced particles [104]. This rise of particle fineness when dropping the temperature is also highlighted by Ghodki and Goswami [102] who have worked on hammer milling of black pepper. Fine particles may be more regular, spherical, and smoother [11,104]. However, this observation is not general, as high roughness and low sphericity can be observed by lowering the temperature [102]. The fine, rough, irregular-shaped particle can lead to a decrease in interparticle distance and an increase in the number of interparticle contact points, which enhances powder cohesion and impairs the powder flowability [102,104,109,110,111].

Chemical Impacts. The decrease in temperature applied to avoid heating during grinding is not without effect, this may lead to an increase in the powder water content. The ambient air steam met the powder’s cold surface and led to water condensation increasing water content and water activity of powders [102]. Water activity increased also when the particle size was reduced as observed by Cid-Ortega and Guerrero-Beltran [107] and Deli et al. [11] on B. senegalensis and *H. sabdariffa* powders obtained by grinding and sieving. Indeed, the reduction in small particle size leads to an increase in the surface area of particles [11,102,104] leading to more exchange with the surrounding air and increased interactions with air humidity, enhancing water absorption, therefore increasing water activity [112].

Furthermore, nutrients and bioactive molecules are more available in fine particles (Table 3) but may be exposed to the environment [11,102,104]. The chemical properties of ground vegetables including hibiscus calyxes vary according to the particle size [11]. Minerals, proteins, and lipids content in *Hibiscus sabdariffa* calyx sieved powders were more important in small particle powders than in large particle powders. This observation is in agreement with studies on *Hypericum perforatum* and *Achillea millefolium* powders, where small particle powders were the richest in minerals [99]. However, the inverse results can be observed for phytochemical compounds. Deli et al. [11] reported also that the smaller the particles, the higher the loss in phytochemicals. This can be explained by the great specific surface area of fine particles, which enhance more exchange with the surrounding environment, leading to phytochemical loss. Nevertheless, this trend depends on the biological material structure and the sensitivity of the bioactive molecules, since in the same study Deli et al. [11] found higher phenols and flavonoid contents in the finest powder of *Boscia senegalesis*, unlike the finest *Hibiscus sabdariffa* powder which contained the lowest quantity of flavonoid and the greatest phenol content. Moreover, these differences may be explained by the sensitivity of phytochemicals to temperature during the grinding process. Studies highlight in addition, the phytochemicals degradation due to the heat during the grinding process [113].

### 3.3. Powder Reconstitution and Biomolecule Extraction

The utilization of powders often requires to put them in an aqueous solution [114,115,116]. The reconstitutability of powders could be an indicator of production process yield and product quality [116,117].

The reconstitution allowing the extraction of soluble molecules is essential to their use. Indeed, bioactive molecules are extracted from materials to be analyzed, identified, and used as functional compounds in other products, as well as medicines. In this sense, this operation could be used to produce beverages from fresh or dried plants, vegetables, or fruits. Powder reconstitution occurs in different stages: wetting, sinking/swelling, dispersion, and solubilization [114].

The first step is the wetting. It is the phenomenon during which the liquid gradually replaces the gas phase at the particle surface after that the liquid was put into contact with the particle surface [116,118,119]. Sinking is the phenomenon during which water diffuses into the particle, increasing its density, which enables the particle to overcome the surface tension at the particle-liquid interface [120,121,122]. Simultaneously, the liquid penetrates the particles and results in an increase in particle size; it is the swelling step. Swelling is also marked by a local increase in liquid viscosity around the particle which would indicate a softening of particle surfaces [121,123,124,125,126]. The third step is the dispersion, which is marked by a division of parent particles (agglomerated or not) into several daughter particles [116] when the interparticle forces (liquid and solid bridges, hydrogen or intraparticle bonds, van der Waals interactions) are broken [121,127]. The final solubilization step corresponds to the disappearance of particle granular structure after the complete solubilization of powder [121,128]. To the best of our knowledge, M’be et al. [10] proposed for the first time a reconstitution mechanism of grinding-induced hibiscus powder. They identified 3 steps after the eventual wetting: swelling, dispersion comprising a first rapid stage of particle dissociation, followed by fragmentation, and finally the quasi-instantaneous molecular solubilization.

#### 3.3.1. Impact of Powder Physicochemical Properties on Reconstitution

Powders have typical characteristics lying in the transformation process and the material properties, which impact their reconstitutability.

The reconstitution properties depend on particle size, shape, density, surface, and structure, and the chemical composition. Authors showed that large, regular, surface-smooth, and less viscous particles due to their smaller contact angles (θ, angle between liquid and solid surfaces) are more wettable [11,119,129,130,131,132]. However, the first two steps (wetting and swelling) could be delayed for hydrophobic particles [121,130] since the particle affinity to water is essential. For example, date powder wettability ranges from 13–27 s [133], and that of commercial milk powders generally ranges from 24 s for skim milk powders to 120 s for whole milk powders [119,130]. The short wetting time of date powders is due to their richness in carbohydrates that are hydrophilic molecules able to enhance wetting in contrast to hydrophobicity caused by lipids located at the particle surface of whole milk powder. Inversely to wetting, smaller particles, due to their high specific surface area, resulting in longer swelling duration as reported by M’be et al. and Fournaise et al. [10,121].

The water uptake upon swelling depends on the particle structure. As demonstrated, porous particles favor the penetration of water inside the particle by capillarity leading to rapid swelling [10,132,134]. In this sense, large and porous particles, and fine particles may yield similar swelling kinetics, hence the importance of powder structure and size. The swelling step depends on the particle’s chemical composition as well as the dispersion and solubilization steps. Indeed, powders rich in polymers such as low molecular weight proteins, carbohydrates (hydrophilic molecules), swell, and solubilize rapidly unlike powders rich in lipids [118,119,128,135,136]. In contrast, even fiber-rich powders due to their high absorption capacity present good swelling capacity, their solubilization could be slowed down to the same rate as for powders rich in insoluble components or crystalline structures. In the latter case, the tight structure of molecules hinders water absorption through the particles [10,101,128,130]. In addition, fine particles due to their great specific surface area accelerate dispersion and solubilization [10,11,121]. The improvement of these two latter steps could be possible in the presence of agglomerate and porous particles. Indeed, absorbed water by capillarity could induce a rapid dispersion.

However, particles having high water activity and water content could limit the reconstitution by clogging pores where water could penetrate [132,134]. This factor relies on the glass transition: above the powder glass transition temperature, powders are in a rubbery state, therefore viscous layers limit the water absorption [132,134], hence the necessity to master the drying and powdering steps.

#### 3.3.2. Impact of Extrinsic Parameters on Reconstitution and Link with Extraction

Reconstitutability also depends on extrinsic factors such as solvent nature, powder/solvent ratio, solvent temperature, and stirring conditions. Under high stirring speed and relatively high temperature, the reconstitution rate is improved, by enhancing the sinking and dispersion steps [128,134,137]. The improved reconstitution rate is on one side due to the increase in shear forces induced by stirring. Moreover, the impact of temperature is explained by a change in particle structure and composition that facilitates the penetration of solvent. In line with these authors [18], Nguyen and Chuyen [46] showed that the rise of the water content (e.g., from 8 mL to 10 mL for 1 g dried calyxes) improved the phenol solubilization but higher water content decreased the biomolecule concentration. Indeed, higher water content cannot increase proportionally the diffusion rate because the solute amount is limited.

It is worth noting that there is a cumulative effect of every reconstitution step on the global reconstitution time and the soluble molecules extraction. Consequently, the extraction yield is directly correlated to the efficiency of the global reconstitution process. As argued by Dupas et al. [132] and Neves et al. [138], wetting is an important driver of the reconstitution since a very long wetting time delays all the reconstitution steps resulting in longer biomolecule extraction. This issue could be overcome at an industrial scale by adding the powder to the liquid media under stirring. Although the swelling could be perceived as the less important step, Mo et al. [139] studying coffee extraction, showed that this step could slow down the extraction. Indeed, it specifically slows down the water diffusion inside the particles and the flow rate. This consequently reduces the biomolecule extraction. About dispersion, M’be et al. [10] evidenced the link between this step and the extraction yield. Their studies on the reconstitution of hibiscus powder reported the improvement of the anthocyanin extraction with the dispersion step. In fact, during the dispersion, the increase of the particle-specific surface area, and the particle/solvent interface improved the anthocyanin release. The last step, the particular or molecular solubilization has a greater influence on extraction. Indeed, a high particle and molecular solubilization rate improved the extraction rate [10,140]. Otherwise, the soluble biomolecules could stay trapped in the insoluble matrix as highlighted by M’be et al. [10]. They hypothesized a potential retention of anthocyanins in fibers included in the ground-produced powder.

The molecule extraction is linked to the success of the reconstitution steps and, therefore, to all physicochemical parameters but also the extrinsic conditions.

The couple temperature/time has an important role in the extraction process. High extraction temperature or long time induces high soluble extraction but also causes instability of food [4,18,81] thus reducing antioxidant activity. Nguyen and Chuyen [46] recommended 90 °C instead of 80 °C and 100 °C, while Chumsri et al. [18] found 50 °C/30 min as optimal parameters. These latter authors focused on phenolic and anthocyanin compounds, while Nguyen and Chuyen [46] worked on the soluble phenolic compounds. By lowering the powder particle size to 11–85 μm, M’be et al. [10] evidenced that the couple temperature/time of the reconstitution media could be reduced to 50 °C/30 s or 20 °C/5 min. The advantage of performing the extraction at 20 °C rather than 50 °C is the improvement of antioxidant activity by the reduction of the risk of degradation of heat-sensitive biomolecules [10].

## 4. Conclusions

Hibiscus calyxes are edible vegetables with attractive natural colorings and have demonstrated health and medicinal benefits. The challenge for the food industry is to be able to respond adequately to consumer demands, reduce food waste, allow availability over a long time, and make the product accessible to all interested parties throughout the world. To this end, several unitary operations have been investigated and successful results have been obtained. Drying is very useful to overcome the problem of seasonality, facilitates transport and thus allows worldwide access to this tropical plant. Complementary methods to drying, such as powder production, have further facilitated the use of hibiscus calyxes by increasing the product-specific surface area. This improves the availability of anthocyanins and polyphenols, the antioxidant activity that is the key indicator of the hibiscus calyx health benefits. However, the main barrier to the application of these processes is the thermo-sensitivity of the anthocyanins, which depends on the parameters of the different processes. In practice, optimums are sought to limit the loss of anthocyanins and polyphenols from hibiscus calyxes and the antioxidant activity reduction. Freeze-drying, although expensive is the least harmful drying method for biomolecules followed by microwave drying. Microwave combined vacuum drying or oven drying could be an alternative to this expensive method, even if the antioxidant activity could be lower than freeze drying. The optimal drying coupled with grinding, cryogenic grinding being the best option, could be not only a method to alleviate this waste issue upon the calyx reconstitution, but also improve the biomolecules accessibility, reconstitutability, and extraction. The main obstacle remains the solubility of the grinding-induced powder. Knowing the biomolecule loss risks during the different operations, it is necessary to evaluate the anthocyanin extraction yield and the antioxidant activity to set up an adequate processing method. Furthermore, new extraction, drying, and powdering technologies are still to be tested to optimize the hibiscus calyx transformation. These includes microwave extraction, desiccant drying, alternation of drying, grinding, and other advanced technologies.

## Figures and Tables

**Table 1:** Drying methods of *Hibiscus sabdariffa* calyxes, their selected parameters, and their efficiency.

Hibiscus Calyx Drying Method	Parameter	Efficiency
Sun-drying [4,12] Sun-drying using a heat pump [12,46]	Variable temperature Non-constant air velocity 4 to 8 days of drying Temperature 40.16 ± 7.24 °C Relative humidity 37.56 ± 15.06% Up to 2 days of drying	Roughly 90% of the reduced water content Drying rate from 0.036 to 0.042 g H_2_O (g DM)^−1^·min^−1^ Water content of 13.05% (DM) Final a_w_ = 0.51 Final a_w_ = 0.51
Oven-drying [12,46]	Temperature 40, 45 °C Relative humidity 31% One day of drying Temperature 55, 65 °C From 3 to 4 h drying Temperature 80 °C/8 h	Drying rate of 0.10 g H_2_O (g DM)^−1^·min^−1^ Water content of 15.5% (DM) Final a_w_ = 0.52 Water content of 12.5% (DM) Water content of 8% (DM)
Dehumidified-air-drying [12]	Room temperature Minimum relative humidity 20% About 20 h of drying	Drying rate of 0.212 g H_2_O (g DM)^−1^·min^−1^ Water content of 16.22% (DM) Final a_w_ = 0.54

**Table 2:** Implementation of the main processing methods of *Hibiscus sabdariffa*.

Drying of Solids	Drying of Liquid	Solid Transformation
Sun-drying Accessible Free Renewable Weather dependent High labor cost Large drying area Oven-drying Easy to handle Controlled conditions Relatively low investment Energy and labor-intensive drying system Longer drying time than spray-drying	Spray-drying Cheaper than freezing Controlled parameters Controlled powder particle size Short time of drying Low efficiency due to caking Freeze-drying Longer drying time High energy consumption High capital cost	Grinding Simple processing Continuous or discontinuous processing Ease of applicability of the product Temperature to be controlled Extraction Simple processing Separation of active ingredients or biomolecules of interest

**Table 3:** *Hibiscus sabdariffa*—based product properties according to the transformation process.

Drying of Solids	Drying of Liquid	Solid Transformation
Sun-drying High water content Biochemical reaction Possible microbial contaminations Loss of biomolecules Oven-drying Browning reaction Not suitable for thermal-sensitive products Loss of biomolecules	Spray-drying Homogenous particle size Stickiness Caking during drying and storage Loss of biomolecules Better solubility Freeze-drying More hygroscopic products than spray-dried products Preservation of initial structure and color Limited nutrient losses Good rehydration capacity due to the formation of porous structure in the product	Grinding Better biomolecules extraction Increase in the specific surface area Maillard reaction risk Risks of biomolecule losses A mix of soluble and insoluble parts of the product Extraction Risks of thermal degradation Risks of formation of compounds harmful to the quality of the extract Instability of biomolecules in the extracts during storage
